# Antidiabetic Effect of Noodles Containing Fermented Lettuce Extracts

**DOI:** 10.3390/metabo11080520

**Published:** 2021-08-06

**Authors:** Soon Yeon Jeong, Eunjin Kim, Ming Zhang, Yun-Seong Lee, Byeongjun Ji, Sun-Hee Lee, Yu Eun Cheong, Soon-Il Yun, Young-Soo Kim, Kyoung Heon Kim, Min Sun Kim, Hyun Soo Chun, Sooah Kim

**Affiliations:** 1 Department of Food Science and Technology, Jeonbuk National University, Jeonju 54896, Korea; ahajisan@korea.kr (S.Y.J.); ej12121@hanmail.net (E.K.); siyun@jbnu.ac.kr (S.-I.Y.); ykim@jbnu.ac.kr (Y.-S.K.); 2 Department of Environment Science & Biotechnology, Jeonju University, Jeonju 55069, Korea; zhm27@foxmail.com; 3 HumanEnos LLC, Wanju 55347, Korea; iyoonseong@daum.net (Y.-S.L.); wandukong5@naver.com (B.J.); 4 Department of Biotechnology, Graduate School, Korea University, Seoul 02841, Korea; wowshl@korea.ac.kr (S.-H.L.); yecheong0127@korea.ac.kr (Y.E.C.); khekim@korea.ac.kr (K.H.K.); 5 Center for Nitric Oxide Metabolite, Department of Physiology, Wonkwang University, Iksan 54538, Korea; mskim@wku.ac.kr

**Keywords:** antidiabetic effect, fermented lettuce extract, GC–TOF-MS, metabolomics, noodles

## Abstract

The aim of the current study was to examine the antidiabetic effect of noodle containing fermented lettuce extract (FLE) on diabetic mice as a pre-clinical study. The γ-aminobutyric acid (GABA) content, antioxidant capacity, and total polyphenol content of the FLE noodles were analyzed and compared with those of standard noodles. In addition, oral glucose and sucrose tolerance, and fasting blood glucose tests were performed using a high-fat diet/streptozotocin-mediated diabetic mouse model. Serum metabolite profiling of mice feed standard or FLE noodles was performed using gas chromatography–time-of-flight mass spectrometry (GC–TOF-MS) to understand the mechanism changes induced by the FLE noodles. The GABA content, total polyphenols, and antioxidant activity were high in FLE noodles compared with those in the standard noodles. In vivo experiments also showed that mice fed FLE noodles had lower blood glucose levels and insulin resistance than those fed standard noodles. Moreover, glycolysis, purine metabolism, and amino acid metabolism were altered by FLE as determined by GC–TOF-MS-based metabolomics. These results demonstrate that FLE noodles possess significant antidiabetic activity, suggesting the applicability of fermented lettuce extract as a potential food additive for diabetic food products.

## 1. Introduction

Diabetes is a common chronic disease that causes high blood sugar levels and affects millions worldwide [1]. For patients with diabetes, various risk factors exist, such as genetics, lifestyle, and unhealthy dietary habits [2]. Thus, they seek to control their blood glucose levels with their diet [3].

Wheat noodles are a popular food in Asian society. However, they are considered as one of the worst foods for diabetes, because their consumption triggers relatively higher fasting glucose concentrations through greater insulin resistance and hyperglycemia [4]. Many diabetics demands new noodles for improving blood glucose level. Therefore, various noodles obtained from mixing food additives [5,6,7], using amylose and resistant starch [8], and subjected to cellulose enzymatic treatment [9,10] have been developed for diabetes in food industry.

Plants such as ginseng, bitter melon, banaba, and garlic [11,12,13,14] are known as effective antidiabetics. Previous in vitro and in vivo studies have reported that fermented foods have antidiabetic properties [15]. Foods such as kombucha (fermented tea beverage), fermented food paste, and fermented soy products could decrease diabetic-associated health consequences. However, to our best knowledge, few studies exist on the antidiabetic effect of lettuce extract or fermented lettuce extract (FLE), even though it has potential health effects including the inhibition of DNA damage, intracellular lipid peroxidation, and proapoptotic pathways, and the regulation of glucose metabolism [16,17,18].

Metabolomics, the study of changes in global metabolites in a particular organism, can be used to reveal the mechanism of natural products [19]. It has been applied to investigate the antidiabetic properties of medicinal plants [20]. Qiu et al. reported the metabolic changes of mulberry branch bark powder in diabetic mice using metabolomics based on gas chromatography-mass spectrometry (GC-MS) [21]. Furthermore, Zhu et al. studied the metabolic profiles of fecal samples from diabetic mice administered with a polysaccharide, a water-soluble β-D-fructan from *Ophiopogon japonicus*, using a GC-MS metabolomics platform [22].

In this study, new wheat noodles containing FLE as food additive were produced and their antidiabetic activity was evaluated in vitro and in vivo. Moreover, the underlying mechanisms altered by the FLE noodles were assessed using metabolomics-based gas chromatography–time-of-flight mass spectrometry (GC–TOF-MS).

## 2. Results and Discussion

### 2.1. Proximate Analysis

The carbohydrate, crude protein, fat, moisture, and ash contents of the standard and FLE noodles were similar (Table 1). The carbohydrate content of standard and FLE noodles was approximately 74%, the highest proportion. The contents of moisture, crude protein, ash, and crude fat of the different noodles were 12%, 9%, 3%, and 1%, respectively. No significant nutritional differences were observed between the standard and FLE noodles, indicating there was no nutritional difference between two type of noodles.

### 2.2. Antioxidant Activities of Noodles

The previous studies have reported that γ-aminobutyric acid (GABA), antioxidants, and polyphenol were critical bioactive compounds in fermented foods responsible for the antidiabetic activity [15]. Therefore, to confirm antidiabetic effect of FLE noodles with fermented lettuce extract, we compared the level of GABA, antioxidant, and total polyphenol standard and FLE noodles.

#### 2.2.1. GABA Analysis

**Figure 1:**
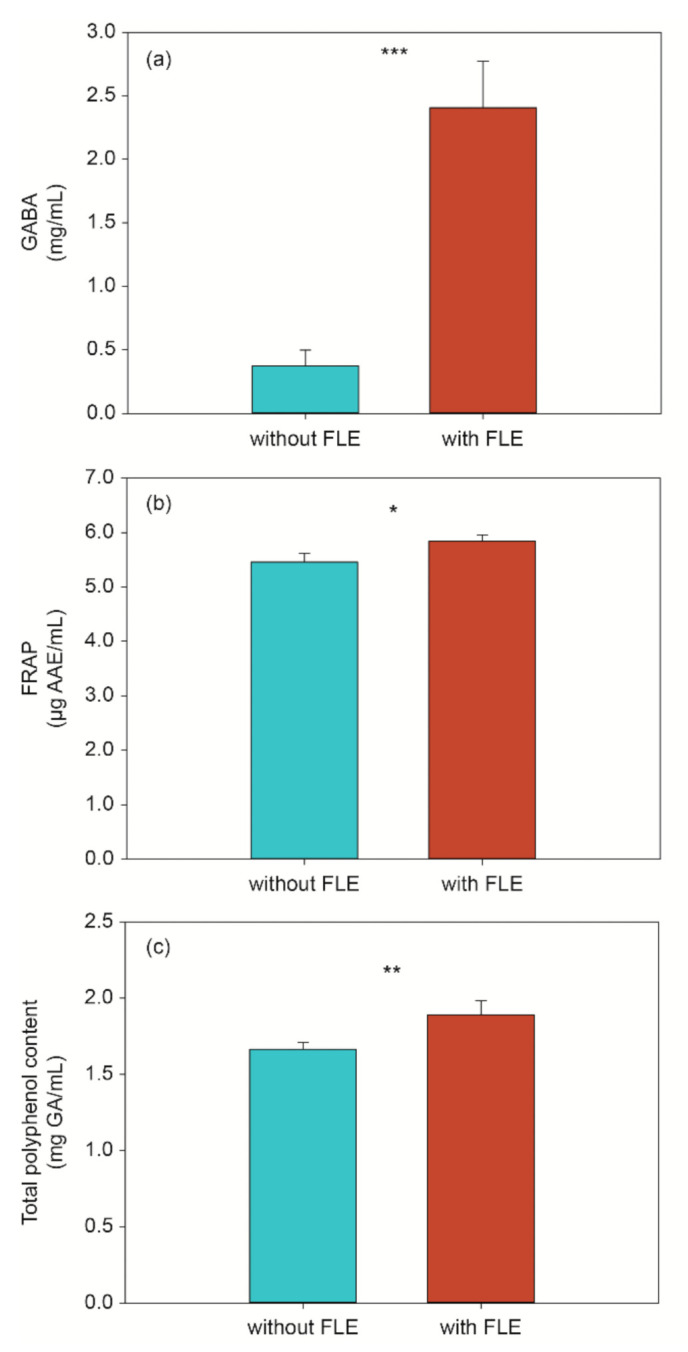
Level of (**a**) GABA, (**b**) antioxidant activity, and (**c**) total polyphenols in standard (without fermented lettuce extract (FLE)) and noodles with FLE (with FLE). The data are shown as the mean ± standard deviation and represent three replicate measurements. Significant difference set as * *p <* 0.05, ** *p <* 0.01, and *** *p <* 0.001.

According to previous studies, GABA could significantly affect the regulation of insulin and glucose homeostasis [23,24]. Therefore, we compared GABA levels in standard and FLE noodles. The GABA levels of FLE noodles were approximately six times higher (2.408 ± 0.363 mg/mL) than those of standard noodles (0.373 ± 0.125 mg/mL) (Figure 1a). Yeap et al. demonstrated the antidiabetic properties of the high level of GABA in fermented mung bean [25]. As with previous results, the high GABA levels in FLE noodles might affect antidiabetic properties.

#### 2.2.2. FRAP Analysis

Antioxidants can prevent the apoptosis of β-cell by oxidative stress and protect its function [26]. Previous studies have shown antioxidants can recover insulin sensitivity and reduce diabetes complications [27,28]. In this study, the antioxidant activity of the different noodles was determined using the ferric reducing ability of plasma (FRAP), which directly measured the ability of antioxidants to reduce Fe^3+^ to Fe^2+^.

A significant difference in antioxidant capacity was observed between the standard and FLE noodles (Figure 1b). The FRAP capacity of FLE noodles was higher than that of standard noodles, suggesting that the FLE noodles might have a higher antioxidant effect and may be an effective additive in the diet of patients with diabetes.

#### 2.2.3. Total Polyphenol Content

Some flavonoids and phenolic acids inhibit the sodium-dependent glucose transporter, delaying the absorption of glucose degraded by digestive enzymes [29]. In addition, studies have reported that they inhibited activity of polysaccharide degrading enzymes and glucose transport [30]. In this study, the Folin–Ciocalteu method was used to determine the content of phenolic acid in the different noodles. As shown in Figure 1c below, the level of total phenolic acid in FLE noodles differs significantly from that in the standard noodles. Similar to the results of a previous study, the total phenolic acid content of the noodles with the fermented extract rose from 1.664 to 1.889 mg gallic acid (GA)/mL [31], indicating that the addition of fermented lettuce extract could improve the antidiabetic effect.

### 2.3. Blood Glucose and Insulin Concentration via In Vivo Digestion of Noodles Using OGTT, OSTT, FBGT, and HOMA-IR

**Figure 2:**
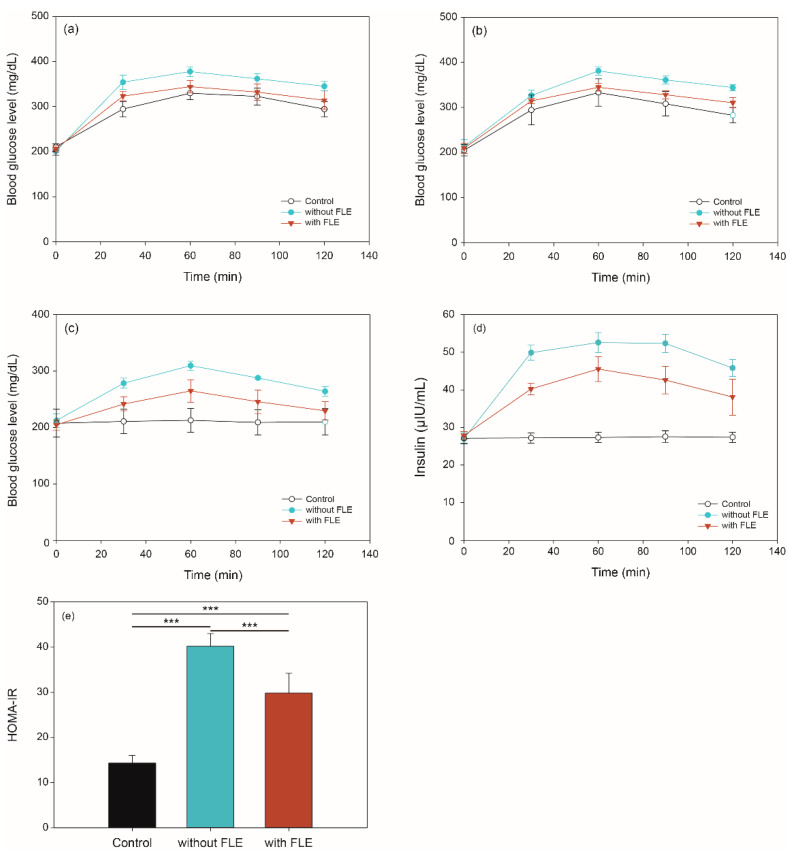
Blood glucose in (**a**) oral glucose, (**b**) sucrose tolerance, and (**c**) fasting blood glucose tests, as well as (**d**) insulin levels and (**e**) HOMA-IR values of mice given distilled water (control), feed standard noodles (without fermented lettuce extract [FLE]), and feed noodles with FLE (with FLE). Significant difference set as *** *p <* 0.001.

For oral glucose tolerance test (OGTT), oral sucrose tolerance test (OSTT), and fasting blood glucose test (FBGT), the blood glucose level in diabetic mice were measured at 0, 30, 60, 90, and 120 min after administering the different feeds. OGTT results showed that the blood glucose levels of mice with standard and FLE noodles were higher than those with control (distilled water) at 30 min (Figure 2a). At 60, 90, and 120 min, the level of blood glucose of mice fed with standard noodles was higher than the control (*p* < 0.01); however, its level did not differ significantly between control and mice fed with FLE noodles. In addition, the blood glucose level of mice fed with FLE noodles was lower than for those fed with standard noodles over 2 h (*p* < 0.05).

The results of the OSTT experiment are shown in Figure 2b. The blood glucose level of mice fed with control and FLE noodles was not significantly different over 2 h. The level in mice fed with standard noodles was higher than the control at 60, 90, and 120 min (*p* < 0.01), however, there is no significant difference between the two groups at 30 min. Moreover, the blood glucose level of mice fed with FLE noodles significantly decreased compared with that of mice fed with standard noodles over 2 h (*p <* 0.05).

Figure 2c shows the changes in mice fasting blood glucose levels over 2 h. When the standard noodles were administered, the blood glucose level of the mice increased over 2 h compared with that in the control (*p* < 0.001). During 90 min, the blood glucose level of mice fed with FLE noodles was higher than that in the control (*p* < 0.05). Furthermore, the blood glucose levels of mice fed with FLE noodles recovered to similar levels as in the control after 120 min.

Insulin level and homeostasis model assessment of insulin resistance (HOMA-IR) were determined to investigate the effect of noodles containing FLE on insulin resistance. As shown in Figure 2d, the fasting insulin levels of mice fed standard or FLE noodles were significantly increased compared with those given distilled water over 2 h (*p* < 0.001). It is noteworthy that the fasting insulin levels in mice fed standard noodles were higher than those in mice feed FLE noodles during 2 h (*p* < 0.001). Moreover, HOMA-IR index in mice that fed on FLE noodles was significantly reduced compared with that in mice that fed on standard noodles (*p* < 0.001) (Figure 2e).

Similar to previous studies, these results revealed that FLE significantly improved the glucose and sucrose tolerances, as well as insulin resistance, while reducing the blood glucose levels in an in vivo model [32,33,34,35,36,37]. These results suggest that the FLE can inhibit digestive enzyme activity and affect glucose absorption, inhibiting the rapid increase of postprandial glucose.

### 2.4. Metabolite Profiling of Serum In Vivo Digestion

#### 2.4.1. Identification of Metabolites

Many studies have discovered biomarkers and investigated the mechanisms of diabetes [38,39,40]. In this study, to understand the metabolism changes caused by FLE in mice with diabetes, serum metabolite profiling was conducted using GC–TOF-MS. A total of 83 metabolites were identified using BinBase, an in-house programmed library, and they were categorized into the following chemical classes: organic acids (21.7%), amino acids (20.5%), sugars (20.5%), fatty acids (18.1%), amines (10.8%), phosphates (3.6%), and miscellaneous (4.8%) (Table 2). These metabolites are major intermediates of various metabolisms, such as glycolysis, amino acid and fatty acid metabolisms, and tricarboxylic acid cycle, and they substantially influence diabetes. Lactate is associated with diabetes metabolism [41].

#### 2.4.2. Metabolite Profiles of the Fermented Extract by PCA and HCA

**Figure 3:**
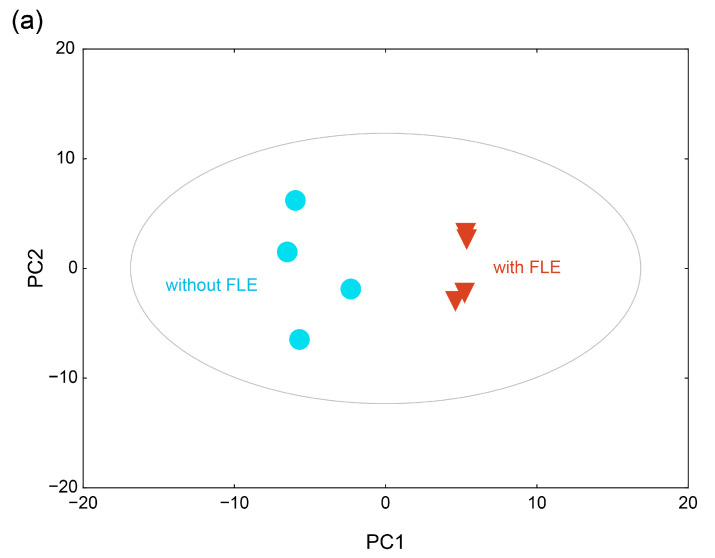

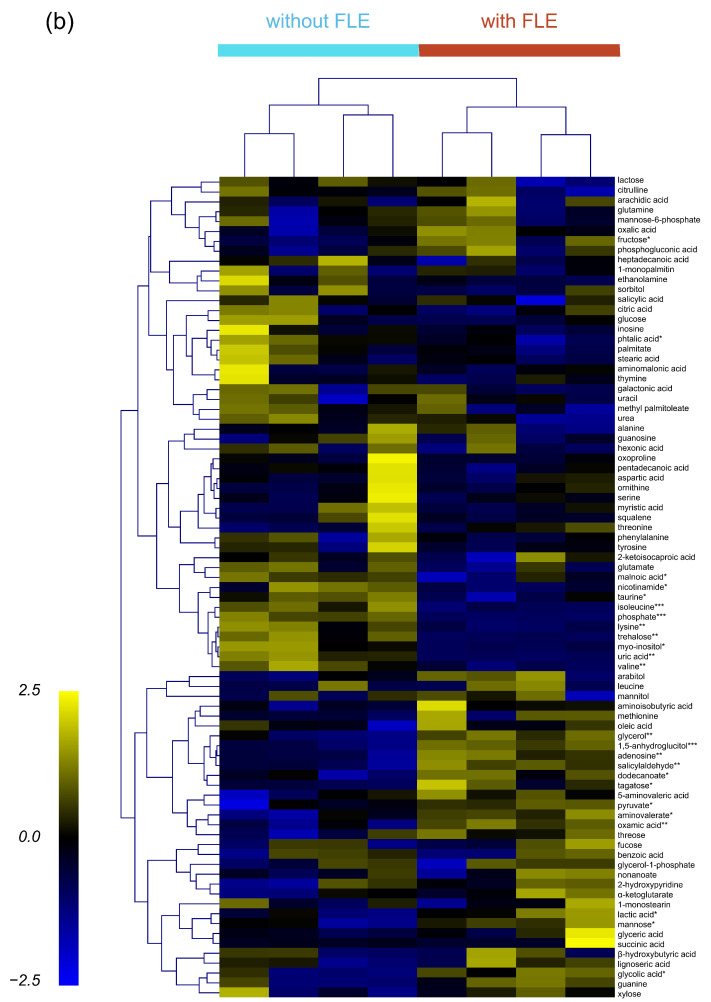
(**a**) Score plot of the principal component analysis (PCA) and (**b**) heatmap of hierarchical clustering analysis for the serum metabolite profiles of mice feed standard (without fermented lettuce extract (FLE)) and noodles with FLE. Significant differences were set as * *p* < 0.05, ** *p* < 0.01, and *** *p* < 0.001.

To investigate the metabolic differences between the group with standard and FLE noodles, principal component analysis (PCA) was conducted. The PCA score plot showed that the metabolite profiles of the groups with standard and FLE noodles were differentiated by principal component (PC) 1 and PC2 (Figure 3a). The variation value (R^2^X) and predictive capability (Q^2^X) of the model were 57.0% and 52.4%, respectively, based on cumulative values of PC1 and PC2, indicating a great quality of explanation and prediction [42]. The loadings of the 20 metabolites with relatively higher values, which represent how the identified metabolites contributed to the PC1 and PC2 generated by PCA, are listed in Table 3. Among the 83 metabolites, 37 metabolites, including oxamic acid, 1,5-anhydroglucitol, adenosine, glycerol, and pyruvate, contributed positively to PC1. However, 46 metabolites, including trehalose, isoleucine, uric acid, valine, and glutamate, contributed negatively to PC1. Forty-seven metabolites, including stearic acid, palmitate, oleic acid, and 1-monopalmitin, contributed positively to PC2, whereas 36 metabolites, including threonine, ornithine, myristic acid, and serine, contributed negatively to PC2.

To cluster and visualize the separation of metabolite profiles between the groups administered with standard noodles and FLE noodles, hierarchical cluster analysis (HCA) was conducted with the Pearson correlation and average linkage using MultiExperiment Viewer (MeV). In the heat map, four biological replicates at each group showed similar metabolite profiles; however, the metabolite profiles differed significantly between groups of standard and FLE noodles (Figure 3b). In addition, the clustering of metabolite profiles between the two groups was caused by certain individual metabolites. For example, the levels of glycerol, pyruvate, and lactic acid were high, whereas those of isoleucine, valine, and lysine were low in the FLE noodles group.

#### 2.4.3. Difference in Metabolite Changes Owing to the Fermented Extract

**Figure 4:**
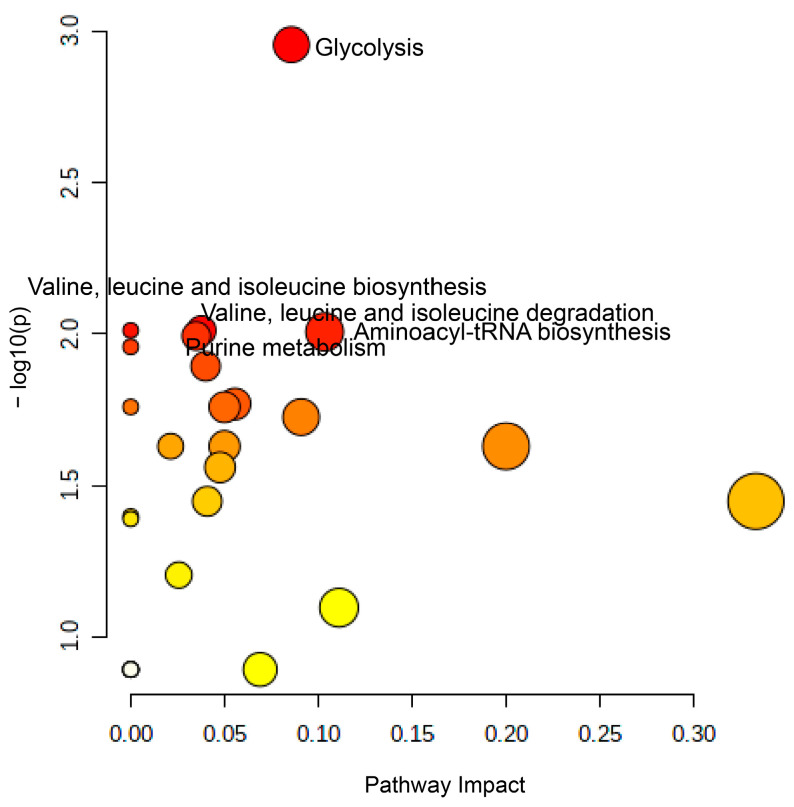
Summary of the pathway analysis results using MetaboAnalyst. The pathway impact (*X*-axis) and −log*p*-value (*Y*-axis) were calculated from the pathway topology analysis and pathway enrichment analysis, respectively. Glycolysis, valine, leucine, and isoleucine metabolism (BCAA metabolism), aminoacyl-tRAN biosynthesis, and purine metabolism were significantly different between mice serum for mice administered with standard and fermented lettuce extract (FLE) noodles at a significance level of *p* < 0.01, with adjusted false discovery rate (FDR) *p-*value < 0.05.

The levels of 12 metabolites differed significantly between the groups with standard and FLE noodles (*p* < 0.05 with false discovery rate (FDR) < 0.5). Of these 12 metabolites, 5 metabolites, including 1,5-anhydroglucitol, adenosine, oxamic acid, and glycerol, exhibited higher levels in FLE noodles group than in the standard noodles group. Conversely, 7 metabolites, including phosphate, isoleucine, uric acid, trehalose, valine, and lysine, had lower levels in the FLE noodles group. We conducted pathway analysis using MetaboAnalyst and founded that these metabolites, which critically contributed to discriminating between standard and FLE noodles, were major intermediates in the glycolysis, valine, leucine and isoleucine metabolism (branched-chain amino acid (BCAA) metabolism) and purine metabolism (Figure 4). The pathway impact and *p*-value were computed form pathway topology analysis and pathway enrichment analysis. The significant pathways were filtered out by setting 0.01 of *p*-value threshold with FDR adjusted *p*-value threshold of 0.05.

Glycolysis, known to control insulin secretion and metabolic functions, can be altered in case of diabetes [43,44]. In this study, the levels of glycolytic pathway intermediates such as lactic acid and pyruvate were higher in the group with FLE noodles. In addition, glucose levels decreased in this group, indicating that glycolysis was faster in diabetic mice administered with FLE noodles. These results agree with previous studies where fermented foods such as food paste, rice bran, soybean, grain foods, tea, and aged black garlic significantly reduced blood glucose levels and increased glucose metabolism [37,45,46,47,48]. This suggests that FLE could trigger glycolysis in patients with diabetes and reduce blood glucose levels, which agrees with the results of in vivo OSTT, OGTT, and FBGT.

Studies have shown that purine metabolism also influences diabetes [49,50,51]; levels of uric acid, inosine, xanthine, hypoxanthine, and AMP are higher in diabetes patients. Our results agree with these reports, in that the abundance of uric acid and inosine was lower in the FLE noodles group, compared with the standard group.

Amino acids have been used as novel biomarkers of diabetes. Previous reports revealed that BCAA metabolism was related to insulin resistance and mammalian target of rapamycin (mTOR) signaling in diabetes [52,53]. Furthermore, clinical studies showed that BCAA level increased in diabetic groups [54]. In this study, the levels of most amino acids decreased in the FLE noodle group, except for glutamine. The levels of BCAAs, such as isoleucine and valine, were especially lower in the FLE noodles group; this agreed with a previous study [54] and reveals that the fermented lettuce extract in noodles could prevent BCAA production in diabetes patients.

Our metabolomics with in vivo results showed that FLE is most effective for regulating diabetes via various mechanisms such as rapid glucose metabolism and the reduction of purine and BCAA metabolism.

## 3. Materials and Methods

### 3.1. Wheat Noodle Preparation

Noodles used in this study were prepared as described in previous studies with slight modifications [55,56]. Briefly, noodles were prepared by mixing 970 g of wheat flour with 30 mL of 7% saline water (standard noodle) or with saline water including 0.5% fermented lettuce extract (FLE noodle). After mixing the flour for 20 min using an electric dough mixer (KMC570, Kenwood, Hampshire, UK), the dough was rolled and extruded using a noodle maker equipped with a rolling mill and slitter machine (Yongma, Daegu, Korea, YMC-103). The raw noodles were dried at 35–40 °C and 70–75% humidity for 10 h. The dried noodles were cut into 30 cm sections and vacuumed-packaged immediately for later use. The noodles of three independent replicates of each group were prepared. The standard and FLE noodles were analyzed in triplicates for carbohydrate, crude protein, fat, moisture, and ash contents using Association of Official Agricultural Chemists methods [57].

### 3.2. FLE

The FLE was produced by HumanEnos LLC (Wanju-gun, Korea) as described in a previous study, with modifications [58]. Fresh lettuce was obtained from a local market. After sterilizing it with ozonized water for 20 h and drying for 24 h, it was powdered to pass through a 4 mm mesh sieve using a cutting mill (KM tech, Icheon, Korea). Under aerobic conditions, *Bacillus subtilis* (KCTC 1201BP) was cultured in the pulverized fresh lettuce mixed with distilled water (1:9, *w*/*v*) at 37 °C for 15–25 days. After fermentation, a supernatant was obtained by ultrafiltration and sterilization, which was used to produce the FLE noodle as described in Section 3.1.

### 3.3. Antioxidant Test

#### 3.3.1. Extraction of Noodles

Noodle extraction was performed as described previously [55]. Noodles (10 g) and 40 mL of 70% ethanol were homogenized for 5 min and incubated at 25 °C for 2 h with agitating at 100 rpm. Then, the mixture was centrifuged at 12,000 rpm for 5 min, and the supernatant was collected and frozen at −20 °C until the analysis of the γ-GABA content, FRAP, and total phenolic content. All experiments were independently repeated three times.

#### 3.3.2. GABA Analysis

The GABA analysis was conducted as described previously with some modifications [59]. For sample derivatization, 3 mL of GABA solution (0–50 mg/mL) for standard or sample for test was mixed with 1.5 mL of 0.5 mol/L NaHCO_3_ and 0.5 mL of 1-fluoro-2,4-dinitrobenzene (FDNB; 0.715 mg/mL) and then incubated at 60 °C for 1 h. After cooling to room temperature, the solution was filtered through a 0.22 μm membrane filter (hydrophilic PTFE; Advantec MFS Inc., Dublin, CA, USA).

Ten microliters of the filtrate was injected into the high-performance liquid chromatography system comprising an LC-20AD pump, an SPE-M20A diode array detector, a CTO-20A oven, a CBM-20A controller, and an SIL-20A autosampler (HPLC; Simazhu, Kyoto, Japan) equipped with a Symmetry C18 column (3.9 × 150 mm, 5 μm). The HPLC fractions were eluted isostatically with 0.5% ammonium acetate aqueous solution and acetonitrile (85:15, *v*/*v*) for 20 min with a flow rate of 1 mL/min. The concentration of GABA was measured using the UV/Vis detector at 360 nm and the column temperature was set at 30 °C.

#### 3.3.3. FRAP Analysis

To compare the antioxidant activity between standard and FLE noodles with fermented lettuce extract, the FRAP assay was performed according to a previously described method, with some modifications [60]. FRAP reagents were prepared by mixing 25 mL of 300 mM acetate buffer (pH 3.6), 2.5 mL of 10 mM TPTZ (2,3,5-triphenyltetrazolium chloride) solution in 40 mM HCl, and 2.5 mL of 20 mM FeCl_3_·6H_2_O solution; the mixture was incubated at 37 °C. Twenty microliters of noodle extract was mixed with 180 μL of the FRAP reagent solution for 30 min under dark conditions. The absorbance value was determined at 593 nm using a UV spectrophotometer (UV-1800, Shimadzu, Kyoto, Japan).

The standard curve of divalent iron ions was obtained from ferrous sulfate, and the reference experiment was conducted with ascorbic acid under the same experimental conditions. Antioxidant capacity was expressed as ascorbic acid equivalents (AAE) per mL of extract solutions.

#### 3.3.4. Total Phenolic Content

The total phenolic content of the noodle extract was determined using the Folin–Ciocalteu method [61]. Briefly, 16 μL of noodle extract was mixed with 60 μL of Folin–Ciocalteu reagent and incubated for 5 min at 25 °C, followed by the addition of 60 μL of 60 g/L sodium carbonate solution. The mixture was reacted for 90 min in the dark. The absorbance was measured at 725 nm using a UV spectrophotometer (UV-1800, Shimadzu), with 50% methanol as the blank. The total phenolic content was expressed in mg GA/g dry weight.

### 3.4. Animal Experiments and Blood Glucose Concentration

Eight-week-old male C57BL/6 mice were purchased from Samtaco (Osan, Korea) and acclimatized for one week before use. In the experimental animal room, the light was controlled at 12 h intervals, the temperature was maintained at 23 ± 2 °C, and humidity was kept at 50–60%. The animal experiment was performed with the approval of the Wonkwang University Animal Experimental Ethics Committee (Approval No. WKU21-44).

#### 3.4.1. Type 2 Diabetes Induction

After mice were fed a high-fat diet (60% calories) for 4 weeks, diabetes was induced in 12 h fasted mice through intraperitoneal injection of streptozotocin (Sigma-Aldrich, St. Louis, MO, USA) in 0.1 M citrate buffer (pH 4.5) at a dose of 120 mg/kg [62,63]. The mice were stabilized for 2 weeks and were excluded with a blood glucose level of 200 mg/dL or less as measured by a portable blood glucose meter (Glucotrend, Roche, Germany). The diabetic mice with fasting blood glucose levels of >200 mg/dL were used for the experiments.

#### 3.4.2. OGTT, OSTT, FBGT, and HOMA-IR

The mice were randomly divided into three groups (i.e., control (distilled water), standard noodles, and FLE noodles with fermented lettuce extract). After 30 min of the administration of distilled water or noodles, the mice were orally treated with 2 g/kg of glucose for OGTT or 2 g/kg of sucrose for OSTT (Table 4a,b). Blood samples were collected from the tail vein at 0, 30, 60, 90, and 120 min and blood glucose levels were measured with a blood glucose meter (Accu-check, Roche, Basel, Switzerland).

For FBGT, the mice were given an oral dose of distilled water or noodles (300 mg/kg) and blood samples were collected from the tail vein at 0, 30, 60, 90, and 120 min to measure blood glucose levels (Table 4c).

The fasting blood insulin levels were determined using a mouse insulin enzyme-linked immunosorbent assay kit (Shibayagi Co., Ltd., Gunma, Japan). To evaluate the degree of insulin resistance, HOMA-IR was calculated with the fasting blood glucose and fasting blood insulin values as follows [64]:

HOMA-IR = fasting blood insulin (μU/mL) × fasting blood glucose (mmol/L)/22.5

All animal experiments involved groups of five replicates and were repeated three times each with all types of noodles.

### 3.5. Metabolite Profiling of Mice Serum

#### 3.5.1. Extraction of Metabolites in the Mice Serum

The extraction of metabolites was performed as described previously with modifications [65]. Briefly, 50 μL of serum was extracted with 250 μL of a solvent mixture, comprising methanol, water, and chloroform (2.5:1:1, *v*/*v*/*v*). The mixture was vortexed for 30 min at 25 °C and then centrifuged at 16,000× *g* for 3 min at 4 °C. Next, 225 μL of supernatant was collected and added to a new clean tube with 200 µL of water. The mixture was vortexed for 10 min at 25 °C and then centrifuged at 16,000× *g* for 3 min at 4 °C. Finally, 200 µL of supernatant was transferred to a new clean tube and dried using a vacuum concentrator (NB-503CIR, N-Biotek, Bucheon, Korea).

For derivatization, the extracted metabolite sample was mixed with 10 μL methoxyamine hydrochloride solution in 40 mg/mL pyridine for 90 min at 30 °C then with 45 μL of *N*-methyl-*N*-(trimethylsilyl) trifluoroacetamide for 30 min at 37 °C. A mixture of fatty acid methyl esters (C8, C9, C10, C12, C14, C16, C18, C20, C22, C24, C26, C28, and C30) was added to the derivatized samples as internal retention index markers that monitored shifts in the retention time during the GC–TOF-MS analysis.

#### 3.5.2. GC–TOF-MS Analysis of Metabolites in Mice Serum

GC–TOF-MS analysis was performed using an Agilent 7890B BC (Agilent Technologies, Santa Clara, CA, USA) coupled with a Pegasus HT TOF MS (LECO, St. Joseph, MI, USA). Derivatized metabolites (1 μL) were injected into an RTX-5Sil MS capillary column (30 m × 0.25 mm, 0.25 μm film thickness; Restek, Bellefonte, PA, USA) with an integrated guard column (10 m × 0.25 mm, 0.25 μm film thickness; Restek) for the separation of metabolites in the samples. The oven temperature was set at 50 °C for 1 min and then ramped to 330 °C at 20 °C/min, at which was held for 5 min. The mass spectra of metabolites were collected in the mass range of 85–500 *m*/*z* at an acquisition rate of 17 spectra/s. The temperature of the ion source and transfer line were set at 250 and 280 °C, respectively, and the ionization mode was set to electron impact at 70 eV. Before starting the analysis, GC–TOF-MS was autotuned using three ions including *m*/*z* 69, 219, and 502 from the perfluorotributylamine spectrum. For quality control, a mixture consisting of 32 pure compounds, including ribitol, purtrescine, alanine, and cholesterol, were analyzed before and after the sample analysis.

The preprocessed GC–TOF-MS data were acquired from the LECO Chroma TOF software (version 3.34; LECO) for detection peaks and deconvolution of mass spectra, followed by the use of BinBase, an in-house database for the identification of metabolites [66]. The peak abundance was normalized using the median of the sum of the peak abundances of identified metabolites in each sample.

### 3.6. Statistical Analysis

Statistica (version 7.1; StatSoft, Tulsa, OK, USA) was used for multivariate and univariate analysis, including the one-way analysis of variance (ANOVA) with post-hoc Tukey’s honestly significant difference test for OGTT, OSTT, and FBGT and PCA for metabolite profiling [67,68]. For visualization and organization of the metabolite profile, HCA was conducted using MeV (Dana-Farber Cancer Institute, Boston, MA, USA) [69]. To evaluate the changes of metabolites and metabolisms between the groups, Student’s *t*-test analysis and pathway analysis with FDR adjusted *p-*value threshold of 0.05 were performed using MetaboAnalyst 5.0 (http://www.metaboanalyst.ca).

## 4. Conclusions

In this study, we demonstrated the antidiabetic property of new FLE noodles created by mixing flour with fermented lettuce extract. The GABA, antioxidant capacity, and total phenol levels in FLE noodles were higher than those in standard noodles. OGTT, OSTT, and FBGT assays showed that the blood glucose levels of mice administered with FLE noodles were lower than those of mice administered with standard noodles. Moreover, compared with standard noodles, when the FLE noodles were administered to mice, their blood glucose levels quickly recovered to the control level. HOMA-IR index revealed that insulin resistance was lower in mice fed FLE noodles than in those given standard noodles. The metabolite profiles differed significantly between the standard and FLE noodles. Based on these metabolomics data, we found higher levels of lactic acid and pyruvate related to glycolysis in the mice administered with FLE noodles. Conversely, lower levels of metabolites associated with purine (such as uric acid) and BCAA metabolisms (such as isoleucine and valine) were observed. This is the first study to investigate the antidiabetic effect of FLE noodles with fermented lettuce extract as a food additive. Therefore, FLE may be used as an additive in a potential diet for patients with diabetes. In further studies, the specific bioactive compound that confers the antidiabetic properties to FLE and human application should be investigated.

## Figures and Tables

**Table 1:** Contents of carbohydrate, crude protein, fat, moisture, and ash in standard (without fermented lettuce extract (FLE)) and noodles with FLE.

Feature (%)	Standard Noodles	FLE Noodles	*p-*Value
Moisture	12.30 ± 0.56	12.10 ± 0.74	0.49
Ash	3.41 ± 0.05	3.44 ± 0.06	0.28
Crude fat	1.25 ± 0.11	1.40 ± 0.02	0.39
Crude protein	9.29 ± 0.58	9.31 ± 0.57	1.00
Carbohydrate	73.74 ± 0.07	73.75 ± 0.19	0.06

Note: Data are shown as the mean ± standard deviation.

**Table 2:** List of identified metabolites by GC–TOF-MS and in-house library.

Chemical Class	Identified Metabolites	*p-*Value	Identified Metabolites	*p-*Value
organic acid	2-ketoisocaproic acid	0.535	glyceric acid	0.200
	5-aminovaleric acid	0.069	glycolic acid	0.024
	α-ketoglutarate	0.105	lactic acid	0.031
	aminomalonic acid	0.416	oxalic acid	0.072
	aminovalerate	0.015	oxamic acid	0.003
	benzoic acid	0.802	phthalic acid	0.032
	β-hydroxybutyric acid	0.610	pyruvate	0.044
	citric acid	0.303	succinic acid	0.440
	galactonic acid	0.337	uric acid	0.001
amino acid	alanine	0.518	methionine	0.083
	aminoisobutyric acid	0.088	ornithine	0.614
	aspartic acid	0.493	oxoproline	0.301
	citrulline	0.663	phenylalanine	0.330
	glutamate	0.062	serine	0.454
	glutamine	0.574	threonine	0.817
	isoleucine	0.001	tyrosine	0.281
	leucine	0.588	valine	0.003
	lysine	0.004		
sugar	1,5-anhydroglucitol	<0.001	mannose	0.029
	arabitol	0.102	myo-inositol	0.011
	fructose	0.047	phosphogluconic acid	0.205
	fucose	0.483	sorbitol	0.289
	glucose	0.154	tagatose	0.033
	glycerol	0.003	threose	0.065
	lactose	0.219	trehalose	0.002
	malonic acid	0.027	xylose	0.527
	mannitol	0.851		
fatty acid	1-monopalmitin	0.789	myristic acid	0.321
	1-monostearin	0.963	nonanoate	0.557
	arachidic acid	0.343	oleic acid	0.244
	dodecanoate	0.021	palmitate	0.121
	heptadecanoic acid	0.165	pentadecanoic acid	0.157
	hexonic acid	0.301	squalene	0.238
	lignoceric acid	0.204	stearic acid	0.239
	methyl palmitoleate	0.153		
amine	adenosine	0.002	nicotinamide	0.013
	ethanolamine	0.131	taurine	0.010
	guanine	0.054	thymine	0.265
	guanosine	0.481	uracil	0.892
	inosine	0.162		
phosphate	glycerol-1-phosphate	0.876	phosphate	<0.001
	mannose-6-phosphate	0.790		
other	2-hydroxypyridine	0.315	salicylic acid	0.407
	salicylaldehyde	0.001	urea	0.088

**Table 3:** Identified metabolites with high absolute loadings on PC1 and PC2.

PC1		PC2	
Metabolites	Loadings	Metabolites	Loadings
oxamic acid	0.955	stearic acid	0.824
1,5-anhydroglucitol	0.931	palmitate	0.760
adenosine	0.899	citrulline	0.736
salicylaldehyde	0.892	oleic acid	0.687
aminovalerate	0.886	1-monopalmitin	0.662
glycerol	0.850	inosine	0.591
tagatose	0.736	ethanolamine	0.583
pyruvate	0.721	phthalic acid	0.555
trehalose	−0.977	threonine	−0.831
phosphate	−0.976	ornithine	−0.823
isoleucine	−0.975	myristic acid	−0.810
uric acid	−0.956	benzoic acid	−0.766
lysine	−0.953	2-hydroxypyridine	−0.752
myo-inositol	−0.896	squalene	−0.740
valine	−0.890	aspartic acid	−0.730
taurine	−0.812	serine	−0.725
glutamate	−0.794	pentadecanoic acid	−0.669
malonic acid	−0.790	nonanoate	−0.661
malonic acid	−0.790	glycerol-1-phosphate	−0.645
phthalic acid	−0.780	α-ketoglutarate	−0.635

**Table 4:** Experimental design to study oral (**a**) glucose and (**b**) sucrose tolerance tests, and (**c**) fasting glucose test of the noodles in a diabetic mice model.

**Group**		**Material**	**Dose**	**Material**	**Dose**
1	Control	Distilled water	-	Glucose	2 g/kg
2	Without FLE	Standard noodle	300 mg/kg	Glucose	2 g/kg
3	With FLE	Noodles with fermented lettuce extract (FLE)	300 mg/kg	Glucose	2 g/kg
**(a)**					
**Group**		**Material**	**Dose**	**Material**	**Dose**
1	Control	Distilled water	-	Sucrose	2 g/kg
2	Without FLE	Standard noodle	300 mg/kg	Sucrose	2 g/kg
3	With FLE	Noodles with fermented lettuce extract (FLE)	300 mg/kg	Sucrose	2 g/kg
**(b)**					
**Group**		**Material**		**Dose**	
1	Control	Distilled water		-	
2	Without FLE	Standard noodle		300 mg/kg	
3	With FLE	Noodles with fermented lettuce extract (FLE)		300 mg/kg	
**(c)**					

## Data Availability

The data presented in this study are available in article.
